# Lymphocyte hydrogen sulfide production predicts intravenous immunoglobulin resistance in children with Kawasaki disease: A preliminary, single-center, case–control study

**DOI:** 10.1097/MD.0000000000013069

**Published:** 2018-11-21

**Authors:** Jing Lin, Huacai Zhao, Fuyong Jiao, Lei Ma, Le Ma

**Affiliations:** a School of Public Health, Xi’an Jiaotong University, Xian; b Department of Urology; c Department of Pediatrics, The Third Affiliated Hospital of Medical College, Xi’an Jiaotong University, Xi’an, China.

**Keywords:** hydrogen sulfide, immunoglobulin, Kawasaki disease

## Abstract

The aim of the study was to identify whether lymphocyte hydrogen sulfide production is a potential biomarker to predict intravenous immunoglobulin (IVIG) resistance in children with Kawasaki disease (KD).

This preliminary, single-center, case–control study conducted between June 2016 and March 2018 in Shaanxi Provincial People's Hospital, 85 children (50 with KD and 35 healthy controls) were included. Laboratory biomarkers were collected from the medical records. All patients with KD received 1 g/kg/d IVIG for 2 days and 30–50 mg/kg/d oral aspirin. The aspirin dose was reduced from 3 to 5 mg/kg/d after body temperature normalized. Plasma hydrogen sulfide levels were detected using sulfide electrode. Lymphocyte hydrogen sulfide levels were detected using the human hydrogen sulfide ELISA kits at the acute stage.

Of 50 patients with KD, 31 and 19 were diagnosed with complete KD (cKD) and incomplete KD (iKD), respectively. Eleven patients with KD were resistant to IVIG treatment. The laboratory biomarker findings and levels of plasma and lymphocyte hydrogen sulfide were significantly different between the patients with KD and control group (*P* < .001). Moreover, lymphocyte hydrogen sulfide production was significantly greater in IVIG-resistant patients than in the IVIG-responsive patients, both in cKD and iKD (*P* = .018 and *P* < .001 respectively). Receiver operating characteristic curve indicated that when the lymphocyte hydrogen sulfide production was >15.285 nmol/min/10^8^ lymphocytes, the sensitivity and specificity for predicting IVIG resistance were 90.9% and 76.9%, respectively.

Lymphocyte hydrogen sulfide production could serve as a predictor of the therapeutic efficacy of IVIG in children with KD.

## 1 Introduction

Kawasaki disease (KD) is an acute systemic vasculitis of unknown etiology that commonly develops in children, and is a leading cause of acquired heart disease among children in developed countries.^[1]^ Treatment with intravenous immunoglobulin (IVIG) and aspirin effectively resolves inflammation and reduces the occurrence of coronary artery lesions in patients with KD.^[2]^ However, nearly 23% of patients with KD may require retreatment and 8% may develop coronary aneurysms despite treatment.^[3]^ Therefore, it is vital to predict IVIG resistance in patients with KD.

Recent studies have shown that hydrogen sulfide is produced enzymatically in all mammalian species including humans and plays a key role in promoting cardiovascular homeostasis and health. Sun et al reported that plasma hydrogen sulfide is a biomarker for predicting coronary artery lesions and IVIG resistance in children with KD^[4]^; however, the number of subjects was small, especially the number of nonresponsive patients with KD with IVIG. Hence, we attempted to validate these findings in a larger sample set. Furthermore, as KD is a systemic inflammatory disease, and the white blood cells may participate in the process, we also analyzed the lymphocyte hydrogen sulfide production in patients with KD and compared the levels between IVIG-resistant and IVIG-responsive patients with KD, both in complete KD (cKD) and incomplete KD (iKD) cases.

## 2 Methods

### 2.1 Subjects

This study was a single-center, case–control study. We enrolled a total of 85 subjects, including 50 children with KD and 35 healthy controls from the inpatient pediatric department of Shaanxi Provincial People's Hospital. All patients with KD received 1 g/kg/d IVIG for 2 days and 30–50 mg/kg/d of oral aspirin. Three days after the body temperature normalized, the aspirin dose was reduced to 3 to 5 mg/kg/d for 8 weeks. Of the 50 patients with KD, 31 were diagnosed with cKD and 19 with iKD. Further, 39 patients with KD were responsive to IVIG treatment, and the remaining 11 were IVIG resistant. All 35 children in the healthy control group exhibited unremarkable medical history and normal findings on physical examination and laboratory tests. All the children and their parents were informed about the purpose of the research and agreed to provide relevant research information. Written informed consent was obtained from their parents, next of kin, or guardians. The study was approved by the ethics committee of Shaanxi Provincial People's Hospital.

### 2.2 General, clinical, and laboratory data

Age and gender were recorded from direct interviews with the patients and their legal guardians. General biomarker data including red blood cell count, white blood cell count, and levels of hemoglobin, blood platelets, and C-reactive protein were collected from the medical records.

### 2.3 Echocardiography

All children with KD typically undergo echocardiography prior to the initial treatment and during the convalescent period. Accordingly, a 2-dimensional color Doppler echocardiography was performed by a cardiologist who had no knowledge of the clinical history of patients. The normal chamber size in standard mode was used, and the wall thickness in systole and diastole, as well as the diameter of the coronary artery were measured. The aortic root, left atrial, left ventricular end-systolic, and left ventricular end-diastolic dimensions, and coronary artery diameter were measured during the echocardiography.

### 2.4 Diagnostic criteria for KD and definition of intravenous immunoglobulin resistance

Kawasaki disease was diagnosed based on the 2004 American Heart Association (AHA)/American Academy of Pediatrics (AAP) guidelines.^[5]^ cKD was diagnosed when subjects had at least 5 of the following 6 major clinical signs: fever that lasted ≥5 days; conjunctival hyperemia in both eyes; changes in the lips and oral cavity; polymorphous exanthema; changes in peripheral extremities; and acute nonpurulent cervical lymphadenopathy. iKD was defined as having ≤4 principal signs, with or without the presence of cardiac lesions.^[6]^ Patients were considered IVIG resistant if they remained febrile 48 hours after the administration of initial IVIG or if they exhibited recrudescent fever.^[7]^

### 2.5 Measurement of hydrogen sulfide plasma concentrations and lymphocyte hydrogen sulfide production

Blood was collected by intravenous puncture into a heparin-coated tube. A total of 0.5 mL plasma was mixed with 0.5 mL antioxidant buffer. Plasma hydrogen sulfide levels were then measured with a sulfide electrode (PXS-270, Shanghai, China). After being rinsed with distilled water and dried, the electrode was immersed in the sample. The electrode potential was recorded when the reading had stabilized. Hydrogen sulfide concentrations were calculated according to the standard curve.^[4]^

In brief, 1 mL of blood was mixed with 1 mL of 0.1 mol/L phosphate-buffered saline, and the mixture was added to lymphocyte separation fluid. The fluid was then centrifuged at 200*g* for 15 minutes at 4°C, and the 2nd layer of lymphocytes was collected. The lymphocytes were washed twice with 0.1 mol/L phosphate-buffered saline and centrifuged at 4000*g* for 10 minutes at 4°C. The cells were counted and the lymphocytes stored in the refrigerator until further use. A total of 1 × 10^8^ lymphocytes were lysed in 900 μL of ice-cold Tris-HCl (50 mmol/L, pH 7.4) and subjected to ultrasound-induced cracking for 15 seconds. Lymphocyte hydrogen sulfide production was then measured using human hydrogen sulfide ELISA kits (Mlbio, Shanghai, China), and hydrogen sulfide production was expressed as unit nmol/min/10^8^ lymphocytes.

### 2.6 Statistical analysis

All data were analyzed using SPSS 13.0 software (SPSS Inc, Chicago, IL). Normally distributed data are presented as mean ± standard deviation and were assessed using *t* tests between 2 groups; continuous data that were nonnormally distributed are expressed as median (interquartile range) and were analyzed by using rank-sum tests. Categorical data are presented as frequency and were compared by using the Chi-squared test. Receiver operating characteristic curve analysis was used to evaluate lymphocyte hydrogen sulfide production for predicting IVIG resistance at the acute stage. A *P*-value of <.05 was considered statistically significant.

## 3 Results

### 3.1 Demographic characteristics and biomarker values between patients with KD and controls

The KD group included 28 boys and 22 girls. The white blood cell count and red blood cell count; levels of hemoglobin, C-reactive protein, and plasma hydrogen sulfide, and lymphocyte hydrogen sulfide production significantly differed between the 2 groups. The plasma hydrogen sulfide levels were significantly lower in the patients with KD, although lymphocyte hydrogen sulfide production was significantly greater in the patients with KD than the controls (*P* < .001, Table 1).

**Table 1:**
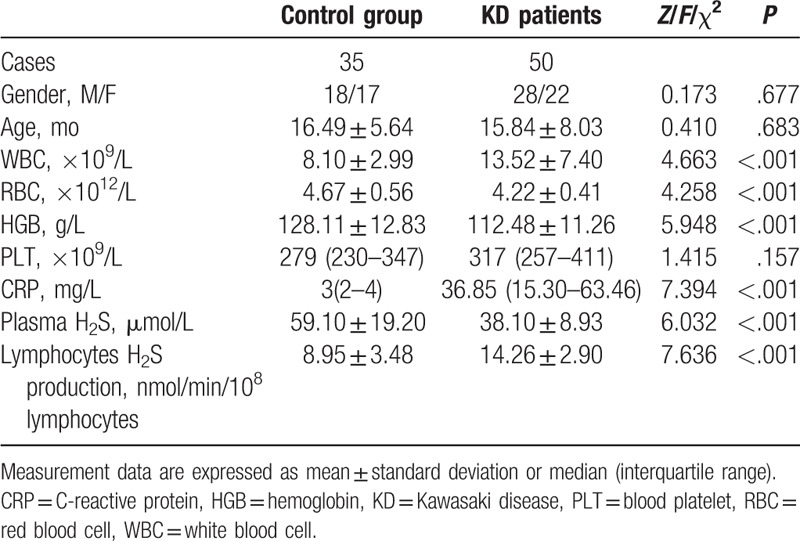
Demographic characteristics and comparison of biomarkers between KD and control groups.

### 3.2 Comparison of laboratory data, plasma hydrogen sulfide levels, and lymphocyte hydrogen sulfide production in patients with cKD and iKD between the IVIG-resistant and IVIG-responsive groups

No significant difference was observed with respect to age, white blood cell count, blood platelets, hemoglobin level, C-reactive protein level, and plasma hydrogen sulfide levels in patients with cKD and iKD between the IVIG-resistant and IVIG-responsive groups. However, lymphocyte hydrogen sulfide production was significantly greater in patients with cKD and iKD in the IVIG-resistant group than in the IVIG-responsive group (*P* = .018 and *P* < .001 respectively, Table 2).

**Table 2:**
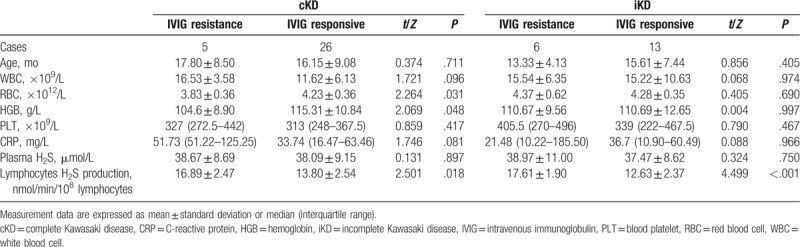
Comparison of laboratory data and lymphocytes H_2_S production between IVIG-resistant group and IVIG-responsive group in patients with cKD and iKD.

### 3.3 Predictive value of lymphocyte hydrogen sulfide production for IVIG resistance at the acute stage in patients with KD

**Figure 1:**
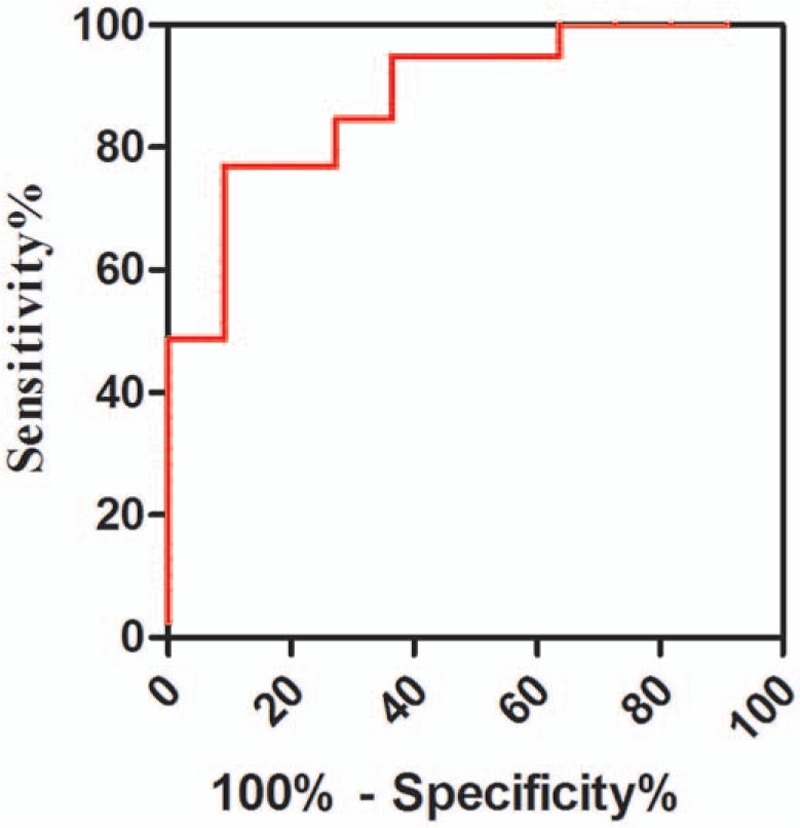
Receiver operating characteristic curve analysis of the predictive value of lymphocyte hydrogen sulfide production for intravenous immunoglobulin resistance in patients with Kawasaki disease at the acute stage. The area under the curve was 88.3% (95% confidence interval, 0.78–0.99), indicating that lymphocyte hydrogen sulfide production was a strong predictor of intravenous immunoglobulin resistance in patients with Kawasaki disease at the acute stage.

The receiver operating characteristic curve shows that the area under the curve was 88.3% (95% confidence interval, 0.78–0.99%; *P* < .001), which suggests that lymphocyte hydrogen sulfide production has a stronger ability to predict IVIG resistance in patients with KD at the acute stage. In fact, when lymphocyte hydrogen sulfide production was >15.285 nmol/min/10^8^ lymphocytes, the sensitivity and specificity for predicting IVIG resistance were 90.9% and 76.9%, respectively (Fig. 1).

## 4 Discussion

In the present study, we found that plasma hydrogen sulfide concentrations were significantly lower in patients with KD than in healthy controls; although lymphocyte hydrogen sulfide production was greater in patients with KD, the extent of the increase was larger in IVIG-resistant patients. Thus, assessment of pretreatment lymphocyte hydrogen sulfide production may serve as a useful predictor of the therapeutic efficacy of IVIG in children with KD, with concentrations of <15.285 nmol/min/10^8^ lymphocytes indicating the potential for clinical improvement after a single IVIG treatment.

Approximately 15% to 25% of patients with KD do not respond to the standard regimen of oral aspirin and IVIG treatment.^[8]^ Hence, it is critical to predict the therapeutic response of patients with KD to the standard treatment regimen in the early phase, which has gained great attention in recent years. Kobayashi et al devised a scoring system by clinical indicators^[9]^; however, Song et al found the models established by these indicators were not precise enough to be clinically applied to some populations.^[10]^ Xie et al^[11]^ and Yang et al^[12]^ reported that some clinical biomarkers may be effective predictors for IVIG resistance; however, as the sensitivity and specificity of these biomarkers were not satisfactory, some may lead to misdiagnosis. In the present study, we found that the plasma hydrogen sulfide concentrations were significantly lower in patients with KD, and there was no significant difference in plasma hydrogen sulfide levels between the IVIG-responsive and IVIG-resistant patients with KD, which was similar to that reported by Sun et al. However, we found that lymphocyte hydrogen sulfide production showed a significant difference between patients with IVIG-responsive KD and patients with IVIG-resistant KD that could serve as a useful predictor of the therapeutic efficacy of IVIG in children with KD.

Recent studies of cardiovascular diseases have shown that the application of physiologic or pharmacologic levels of hydrogen sulfide can reduce myocardial damage, protect blood vessels, limit inflammation, and regulate blood pressure.^[13–16]^ In fact, its protective effect against inflammation is one of the earliest proposed beneficial physiologic effects of hydrogen sulfide.^[15]^

Several studies have shown that hydrogen sulfide stimulates endothelial cell proliferation and migration either by further developing endothelial cell or by developing primary endothelial cells.^[14]^ Hydrogen sulfide participates in vascular endothelial growth factor signaling, and hence, a decrease in plasma hydrogen sulfide levels may induce a reduction in endothelial cell proliferation and endothelial necrosis, both of which are pathological changes observed in patients with KD. Hydrogen sulfide could also hinder leukocyte adhesion by inhibiting leukocyte “rolling” and hence, prevent a firm adhesion to the endothelium. Hydrogen sulfide has been shown to significantly inhibit the expression of leukocyte adhesion molecules.^[17,18]^ Thus, a decrease in plasma hydrogen sulfide levels may enhance leukocyte adhesion. Coincidentally, in patients with KD, initial neutrophil infiltration of the coronary arteries, with necrosis of the arteries beginning at the luminal endothelium, is observed in the first 1 to 2 weeks of the illness. Hence, a decrease in plasma hydrogen sulfide levels may be associated with leukocyte adhesion or neutrophil infiltration.

It remains unclear why IVIG is beneficial in KD. One possibility is that IVIG can reduce tissue inflammation and excessive immune activation by binding to blood mononuclear cells, endothelial cells, or platelet surface Fc receptor, and thereby prevent immune-mediated injury of the intimal surface.^[19]^ However, the mechanism responsible for the increased lymphocyte hydrogen sulfide production in IVIG-resistance KD is unknown. We speculate that the potential reason lies in the imbalance of lymphocyte hydrogen sulfide and metalloproteinase-9 (MMP-9).

The MMPs are a family of zinc-dependent proteinases mostly derived from white blood cells. IVIG has also been shown to reduce the levels of MMP-2, MMP-3, and MMP-9, which are significantly elevated in patients with KD.^[20,21]^ Reducing the MMP expression level played a protective role in the subendothelial basement membrane and intimal elastin. The increased lymphocyte hydrogen sulfide may cause over-activation of the immune cells, and induce large quantities of inflammatory cytokines such as tumor necrosis factor-α, interleukin (IL)-1β, and IL-6 that cause the increment of MMP-9. When the IVIG failed to reduce the level of MMP-9, IVIG-resistance may occur. However, the exact relationship between lymphocyte hydrogen sulfide production and MMP-9 needs further exploration to confirm.

Based on our findings, we believe that lymphocyte hydrogen sulfide production is a potentially useful biomarker of IVIG resistance at the acute stage. Moreover, hydrogen sulfide is continuously secreted and can be easily detected. In addition, only 1 mL of blood is required for detection. Hence, the ease and relatively low cost of detection are advantages of using lymphocyte hydrogen sulfide production as a biomarker for predicting the outcome of coronary artery injury.

The present study has certain limitations. The number of patients studied was relatively small, and all specimens were collected from only one hospital. Nevertheless, considering the occurrence rate of IVIG resistance, it was challenging to collect a large number of samples in a short term. We hope that our findings will lead to further research and clinical trials from multiple centers to validate these results and elucidate the possible mechanism of increased lymphocyte hydrogen sulfide production in IVIG-resistant patients.

## Acknowledgment

The authors thank all the medical staff of the Department of Pediatrics of Shaanxi Provincial People's Hospital for their strong support on the data collection.

## Author contributions

**Data curation:** Lei Ma, Le Ma.

**Formal analysis:** Jing Lin, Le Ma.

**Investigation:** Huacai Zhao, Fuyong Jiao, Lei Ma.

**Methodology:** Huacai Zhao, Fuyong Jiao, Le Ma.

**Project administration:** Jing Lin, Huacai Zhao, Le Ma.

**Resources:** Fuyong Jiao.

**Software:** Lei Ma.

**Supervision:** Jing Lin.

**Validation:** Fuyong Jiao.

**Writing – original draft:** Jing Lin, Huacai Zhao, Lei Ma.

**Writing – review & editing:** Jing Lin, Huacai Zhao, Fuyong Jiao, Le Ma.
